# Dual‐stream algorithms for dementia detection: Harnessing structured and unstructured electronic health record data, a novel approach to prevalence estimation

**DOI:** 10.1002/alz.70132

**Published:** 2025-05-05

**Authors:** Taya A. Collyer, Ming Liu, Richard Beare, Nadine E. Andrew, David Ung, Alison Carver, Jenni Ilomaki, J. Simon Bell, Amanda G. Thrift, Walter A. Rocca, Jennifer L. St Sauver, Alicia Lu, Kristy Siostrom, Chris Moran, Helene Roberts, Trevor T.‐J. Chong, Anne Murray, Tanya Ravipati, Bridget O'Bree, Velandai K. Srikanth

**Affiliations:** ^1^ National Centre for Healthy Ageing Frankston Victoria Australia; ^2^ Peninsula Clinical School, School of Translational Medicine Monash University Frankston Victoria Australia; ^3^ Developmental Imaging Murdoch Children's Research Institute Melbourne Victoria Australia; ^4^ Centre for Medicine Use and Safety, Faculty of Pharmacy and Pharmaceutical Sciences Monash University Parkville Victoria Australia; ^5^ Department of Medicine, School of Clinical Sciences at Monash Health Monash University Clayton Victoria Australia; ^6^ Division of Epidemiology, Department of Quantitative Health Sciences Mayo Clinic Rochester Minnesota USA; ^7^ Department of Neurology Mayo Clinic Rochester Minnesota USA; ^8^ Women's Health Research Center Mayo Clinic Rochester Minnesota USA; ^9^ Department of Geriatric Medicine Peninsula Health Frankston Victoria Australia; ^10^ Department of Neurology Monash Medical Centre Clayton Victoria Australia; ^11^ Turner Institute for Brain and Mental Health, School of Psychological Sciences Monash University Notting Hill Victoria Australia; ^12^ Division of Geriatrics, Department of Medicine Hennepin HealthCare, Berman Centre for Outcomes and Clinical Research Hennepin Healthcare Research Institute Minneapolis Minnesota USA; ^13^ Department of Neurology University of Minnesota Minneapolis Minnesota USA

**Keywords:** big data methods, dementia prevalence, electronic health record data, natural language processing, predictive algorithms

## Abstract

**INTRODUCTION:**

Identifying individuals with dementia is crucial for prevalence estimation and service planning, but reliable, scalable methods are lacking. We developed novel set algorithms using both structured and unstructured electronic health record (EHR) data, applying Diagnostic and Statistical Manual of Mental Disorders criteria for dementia case identification.

**METHODS:**

Our cohort (*n* = 1082) included individuals aged ≥ 60 with dementia identified through specialist clinics and a comparison group without dementia. Clinicians from Australia and the United States informed predictor selection. We developed algorithms through a biostatistics stream for structured data and a natural language processing (NLP) stream for text, synthesizing results via logistic regression.

**RESULTS:**

The final structured model retained 16 variables (area under the receiver operating characteristic curve [AUC] 0.853, specificity 72.2%, sensitivity 80.6%). NLP classifiers (logistic regression, support vector machine, and random forest models) performed comparably. The final, combined model outperformed all others (AUC = 0.951, *P* < 0.001 for comparison to structured model).

**DISCUSSION:**

Embedding text‐derived insights within algorithms trained on structured medical data significantly enhances dementia identification capacity.

**Highlights:**

- Algorithmic tools for detection of individuals with dementia are available; however, previous work has used heterogeneous case definitions which are not clinically meaningful, and has relied on proxies such as diagnostic codes or medications for case ascertainment.
- We used a novel, dual‐stream algorithmic development approach, simultaneously and separately modeling a clinically meaningful outcome (diagnosis of dementia according to specialized clinical impression) using structured and unstructured electronic health record datasets.
- Our clinically grounded case definition supported the inclusion of key structured variables (such as dementia International Classification of Disease codes and medications) as modeling predictors rather than outcomes.
- Our algorithms, published in detail to support validation and replication, represent a major step forward in the use of routinely collected data for detection of diagnosed dementia.

## 1 BACKGROUND

Approximately 50 million people worldwide live with dementia, a number expected to triple by 2050.^1^ As many nations confront aging population demographics, accurate prevalence estimates are essential for planning medical care and support services for people living with dementia, now and into the future.^2^, ^3^ While surveys and cohort studies provide prevalence snapshots for particular groups, national, routinely collected health data assets hold unrealized promise for monitoring dementia prevalence at scale.^4^ Unfortunately, accurate recognition of individuals diagnosed with dementia via routinely collected data is challenging. Typically, scholars and governments rely on three proxy indicators of diagnostic status in administrative data; hospital‐generated International Classification of Disease (ICD) codes for dementia, documented use of anticholinesterase inhibitors, and death certificate data. However, when validated against specialist clinician diagnosis, sensitivity for dementia diagnosis using ICD codes is low,^5^ not all people diagnosed with dementia are prescribed medications, and death certificate reporting is unreliable^3^ leading to systematic underestimation of prevalence and disease burden.^3^

Additionally, the overwhelming majority of published tools for algorithmic detection of dementia do not aim to detect *diagnosed* dementia.^5^, ^6^, ^7^ Many do not use clinically meaningful case definitions^5^, ^8^ (or misapply terms with established definitions such as “probable” dementia),^7^ and/or do not include a group without dementia,^7^, ^8^ severely limiting usefulness in discriminating between people with and without a diagnosis. This reflects the general state of evidence regarding dementia prevalence, noted in the Global Burden of Disease report to suffer from “extreme heterogeneity in case‐ascertainment.”^3^

Against this backdrop, artificial intelligence (AI) techniques such as natural language processing (NLP) applied to vast unstructured (text) data within electronic health record (EHR) systems hold unrealized promise for improving capture of diagnosed dementia.^7^. In practice, complexities in extracting large volumes of text from proprietary EHR databases have limited application of these techniques.^9^ Additionally, progress is hampered by poor integration of siloed research communities, developing clinical algorithms via NLP‐based and biostatistical methods.^10^

As a key step toward.(1) maximizing gain from structured and unstructured EHR data in algorithm development, and (2) improving routine, population estimation of dementia prevalence, we aimed to develop a novel suite of algorithms using both structured and unstructured sources, to reliably identify individuals with diagnosed dementia. Our central hypothesis was that an algorithm combining insights from structured and unstructured, routinely collected information would outperform algorithms developed using either data source alone.

## 2 METHOD

### 2.1 Study setting

Data were sourced via the National Centre for Healthy Ageing (NCHA) Data Platform,^11^ an internally linked, curated, research‐ready warehouse containing data for > 1 million Australians over 10 years (2014–2024).^12^ Records are routinely collected within Peninsula Health, the sole public hospital provider in the Frankston–Mornington Peninsula region (Victoria, Australia) comprising four hospitals and > 10 outpatient clinics. This water‐bounded region has a population of ≈ 310,000, with wide socio‐economic diversity and an aging population (≈ 29% aged > 60 years).^13^ Peninsula Health offers diagnosis and care of dementia via a specialist outpatient Cognitive Dementia and Memory Service (CDAMS).

**Research in context**

1. **Systematic review**: A PubMed search for “dementia” and “electronic health records” with terms like “natural language processing” or “algorithm” yielded 22 studies. Most relied on structured data or partial text (e.g., discharge summaries), missing many dementia‐related terms. Almost all studies lacked robust, clinically grounded case definitions, relying instead on proxies such as diagnostic codes or medications for ascertainment.
2. **Interpretation**: Sensitive algorithms for detecting diagnosed dementia from electronic health records likely require gold‐standard diagnostics for development, and comprehensive structured and unstructured data. Our algorithms represent a major advance in use of routinely collected data for dementia detection and monitoring.
3. **Future directions**: This article offers a framework for efficient dementia monitoring and clinical detection through biostatistical and machine learning methods. Our dual‐stream, multidisciplinary approach could aid detection of other under‐recognized neurological conditions in health care. Additionally, this framework could help identify individuals with high likelihood of undiagnosed dementia, supporting clinical care and diagnostic pathways.

### 2.2 Cohort assembly and outcome definition

**FIGURE 1:**
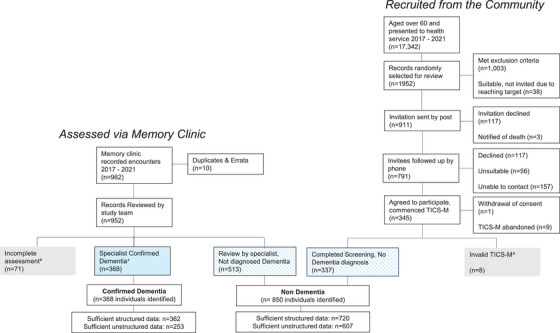
Study cohort recruitment. TICS‐M, Telephone Interview for Cognitive Status–Modified.

Individuals with EHR data available and a confirmed dementia diagnosis (“confirmed dementia,” Figure 1) were identified from 962 consecutive attendances at CDAMS (2017–2021). Specialist correspondence arising from cognitive assessment was reviewed, and the diagnosis communicated to patients’ usual primary care doctor was recorded. The date of the first letter communicating the diagnosis was considered the diagnosis date.

A comparison group aged > 60 years confirmed to not have dementia was established. EHR data at time of recruitment (June 2020) were reviewed to confirm eligibility. Individuals were ineligible if they had: (1) no EHR data available; (2) mention of neurological disorder including dementia, stroke, severe mental health illness (schizophrenia, psychosis, depression), hearing loss, or substance abuse in past medical history; (3) documented CDAMS clinic attendance; or (4) were living in or approved for residential aged care (indicating high risk of diagnosed dementia). Eligible individuals who agreed to participate, and confirmed never being diagnosed with dementia, were assigned to the “non‐dementia” group (Figure 1). To characterize the cognition of those without dementia recruited from the community, the Telephone Interview for Cognitive Status‐Modified (TICS‐M)^14^ test was administered by phone. Those who attended CDAMS but diagnosed as not having dementia were also allocated to the non‐dementia group.

### 2.3 Model development framework

The classification problem consisted of ascribing individuals’ status as either being “likely” or “unlikely” to have received a specialist diagnosis of dementia.

**FIGURE 2:**
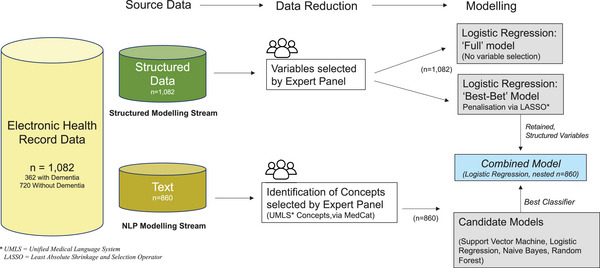
Model development workflow. NLP, natural language processing.

Algorithms were developed in parallel, via three work streams (Figure 2) proceeding via Steyerberg's 7‐step framework,^15^ modified to accommodate data science workflows (Figure S1 in supporting information). Stream 1 was a biostatistical approach^15^, ^16^ using structured EHR data elements available within the EHR (“structured modeling stream”). Stream 2 used a data science approach, applying NLP to unstructured (text) parts of the EHR for the same cohort (“NLP.stream”). Stream 3 (“combined.stream”) combined classification of dementia status from Stream 2 with the final model from Stream 1, to examine additive value of unstructured data.

A panel of clinical experts guided predictor selection in Stream 1 and concept selection/text annotation in Stream 2. The panel comprised six clinicians from Australia and the United States (two neurologists, four geriatricians), and one Australian health information specialist with experience in clinical coding. For patients recruited via CDAMS, EHR data dating 2 years prior to discharge from CDAMS to that most recently available were included for model development. After calculating the average number of years included for those recruited via CDAMS, data for the same duration were included for those recruited from the community.

### 2.4 Data acquisition

Structured data were sourced from eight clinical systems comprising demographics, inpatient admissions, emergency presentations, pharmacy, pathology, imaging, community health, and outpatient appointments (Table S1 in supporting information). Data elements included date/type of admission, principal/secondary diagnosis codes (International Statistical Classification of Diseases and Related Health Problems, Tenth Revision, Australian Modification.[ICD‐10‐AM]), medication lists, procedure codes (e.g., imaging), clinic attendance, birth date, discharge destination, usual residence, postcode, and sex. Data completeness and quality were maximized by combining data across systems. For example, comorbidities were coded using combinations of diagnosis and medication datasets, for conditions known to be under‐recorded using ICD‐10 codes^17^ and missing items were supplemented from secondary datasets when possible. In cases of conflicting demographic data (postcode, age, or sex) the most common entry (mode) was accepted.

The study corpus for the NLP stream consisted of admission and discharge summaries, medical and nursing progress notes, and allied health assessments stored in inpatient EHR systems (Cerner Millennium). All inpatient electronic documents associated with the study cohort created between relevant dates were retrieved from the NCHA Data Platform. CDAMS was external to this platform, and only used for diagnostic status. Automated extraction of documents and pre‐processing of text was performed using CogStack^18^ as follows: (1) extraction of documents and relevant metadata from source systems, (2) consolidating large documents stored as fragmented parts, (3) decompression,; (4) text extraction from formatted files (e.g., portable document format [pdf]), and (5) distributing documents for storage and post‐processing.

### 2.5 Model development (see Box 1)

In Stream 1, structured modeling, model development using structured data proceeded according to the Statistical Analysis Plan for a broader project aimed at refining methods for estimating dementia prevalence,^19^ informed by published literature and biostatistical guidelines for predictive model development.^15^, ^16^, ^20^ “Age only” and “base” logistic regression models (Box 1) acted as performance benchmarks for more complex models.

**Box 1 Modeling streams**

**Structured modeling stream**

**Performance benchmark**:

- Age‐only model:

*Age alone included as a predictor*

- Base model

*Included; Age, sex, social deprivation score, presence of dementia ICD‐10 code*

**Classifiers**:

- Full model

*Included expert‐rated variables, organized into Tiers (1–3)*

- Least Absolute Shrinkage and Selection Operator (LASSO)‐penalized model

*Full model, subjected to LASSO penalization*

**NLP (unstructured data) modeling stream**

Model inputs: person‐level feature vector consisting of Term Frequency Inverse Document Frequency scores for each concept identified by clinical and coding experts.

**Performance benchmark**:

- Naïve model (presence of any Unified Medical Language System (UMLS) concept without negation)

**Classifiers**:

- Logistic regression
- Naïve Bayes
- Support vector machine
- AdaBoost
- Random forest

Neurology panel members identified 163 keywords (corresponding to 108 UMLS concepts), 131 of which were present in available records.

Geriatric medicine panel members identified 457 keywords (corresponding to 229 UMLS concepts), 265 of which were present in available records.

Medical coder identified 68 keywords (corresponding to 36 UMLS concepts), 37 of which were present in available records

**Combined modeling stream**

The combined logistic regression model incorporated all variables retained by LASSO, plus the (continuous) prediction of dementia diagnosis probability output by the random forest NLP model.

*Note*: See Supporting information for logistic regression equations and http://github.com/NCHADataPlatform/dementia_diagnosis_detection for final NLP models.

Abbreviations: ICD, International Classification of Disease; LASSO, least absolute shrinkage and selection operator; NLP, natural language processing; UMLS, unified medical language system.

To construct the full model we identified 103 potential predictors from the literature (Table S2 in supporting information). To select variables for inclusion, the clinician panel completed a data reduction survey, rating each variable's predictive value from 1 (not important at all) to 5 (most important). Members could also suggest variables not contained in the survey. Based on clinician ratings, variables were organized into tiers (Tier 1, rated ≥ 4/5 by 100% of members; Tier 2, majority scored ≥ 4/5; and Tier 3, others) and sequentially included in logistic regression models until the number of supported predictors was reached, subject to observed *R* ^2^.^20^, ^21^ To produce the simplest, final model, the full model was subject to least absolute shrinkage and selection operator (LASSO).penalization.^16^

We conducted two sensitivity analyses. First, we excluded those scoring in the “below average” band or lower on TICS‐M, relative to published population norms^14^ after adjusting for age, sex, and education. Second, we evaluated performance of algorithms developed from structured data among those with sufficient unstructured data.

For sample power estimation, we applied the Riley method using pmsampsize.^21^ With initial projected sample size of 590 (63% with dementia), 17 candidate predictors were well supported with Nagelkerke *R* ^2^ > 0.22.^19^

For Stream 2, unstructured/NLP modeling, we used NLP techniques comprising (1) data normalization using automated concept annotation; (2) concept filtering (data reduction) based on expert input; (3) descriptor vectors derived from counts of filtered concepts; and (4) statistical and machine learning classifiers. This approach is conceptually similar to the structured modeling stream, supports interrogation of all steps, and is compatible with our sample size and data sensitivity. Unsupervised concept annotation was achieved using the Medical Concept Annotation Tool (MedCAT), based on the Unified Medical Language System (UMLS).^22^ Clinical expert–generated words and phrases were used to select UMLS concepts for classifiers assigning dementia status (likely/unlikely), as follows.

Because MedCAT has an unsupervised approach to annotation, domain experts in medical coding (E.L.), specialist neurology (H.R.), and geriatrics (A.L., K.S.) identified a subset of concepts from the UMLS judged likely to be predictive of the presence of diagnosed dementia. This subset was provided to the classifier phase and is analogous to the expert‐driven data‐reduction process in the structured stream. The experts generated lists of keywords and phrases in response to the following questions: Medical coding: “What words/phrases would prompt you to search for a dementia diagnosis when coding a patient?”; and Neurology/Geriatrics: “What words/phrases would you expect to record when seeing a patient with dementia or diagnosing a patient with dementia?” These were converted to concepts, and groups of concepts defined in the UMLS^22^ using a corpus consisting of up to 10 documents from the EHR, containing examples of each of these words/phrases not involving the study cohort. This sample corpus provided further clinical context for MedCAT when assigning concepts to words/phrases in the EHR. See Table S3 in supporting information for further details regarding corpus characteristics.

A keyword search was included as a performance benchmark. Statistical models (support vector machine [SVM], logistic regression, Naïve bayes, AdaBoost, random forest) were then explored. Input to each model was a person‐level feature vector consisting of term frequency inverse document frequency (TF‐IDF) scores^23^ for each concept and negated concept from the expert‐informed set. The TF‐IDF score captures concept importance by adjusting for the fact that words occur more frequently in some documents than others. We used the scikit‐learn^24^ implementation of statistical classifiers, with default hyper‐parameters.

For Stream.3,.combined modeling, after examining performance of NLP models, the binary prediction from the best‐performing classifier was extracted, and re‐purposed as a predictor within the LASSO‐penalized (structured) model. We understood this added predictor as reflecting insight derived from complex patterns within clinical text.

### 2.6 Model performance

Performance was measured by sensitivity/recall, specificity, positive and negative predictive value, proportion of individuals correctly classified (accuracy), and area under the receiver operating characteristic curve (AUC). AUCs were corrected for optimism,^16^ calibration plots were produced, and performance metrics estimated via bootstrap, with 200 repetitions for structured models and 30 for NLP models. To reflect clinical preference for false positives over false negatives in this clinical context, performance was scrutinized at varying classification thresholds and thresholds maximizing sensitivity while maintaining acceptable accuracy were selected (sensitivity optimization).

## 3 RESULTS

### 3.1 Study cohorts

In the confirmed dementia group, on review of notes from CDAMS, 10/962 (1%) were duplicates (Figure 1). Of those remaining, 368 (39%) were diagnosed with dementia, 513 (54%) received an alternative diagnosis, and 71 (7%) had incomplete assessment (no diagnosis). Thirty‐eight ambiguous diagnostic terms were sent for additional review. Of these, 16 were classified as dementia (Table S4 in supporting information).

To create the non‐dementia group, of 17,342 individuals aged ≥ 60 years identified in the NCHA Data Platform, 1952 were randomly selected for review to confirm suitability. This number was selected after modeling backward from recruitment targets, anticipating response rates and ineligibility. Of these, 911 met inclusion criteria, and 345 (38%) agreed to participate. Eight interviews (2%) could not be completed under valid conditions. The remaining 337 (216 scoring in the above average band or higher, 121 scoring below average but having no dementia diagnosis) and those assessed by specialists at CDAMS but not receiving a dementia diagnosis formed the non‐dementia group (337 + 513 = 850, Figure 1).

Of these 1218 individuals, 1082 had sufficient structured data and 960 individuals (253 with dementia) had sufficient unstructured data for algorithm development (Figure 1, Table 1). Variation in data availability occurred due to (1) some patients accessing only outpatient health services (not inpatient care) which use data systems other than Cerner (6/368 with dementia, 92/513 non‐dementia diagnosis at CDAMS, and 10/216 recruited from the community) and (2) staged implementation of Cerner systems across the study period, resulting in patients without text data (e.g., Cerner was implemented in the emergency department, prior to inpatient settings).

**TABLE 1:** Cohort characteristics.

		Non‐dementia (*n* = 720)			
	Confirmed dementia (*n* = 362) Specialist confirmed: CDAMS	Non‐dementia: CDAMS	≥ Average on TICS‐M^a^	< Average on TICS‐M^a^	Total cohort with sufficient data for structured algorithm development^b^
	*N* = 362	*N* = 421	*N* = 206	*N* = 93	*N* = 1082
Age, mean (SD)	79.9 (7.3)	77.5 (8.2)	71.7 (6.2)	73.7 (7.8)	76.9 (8.1)
Female sex	211 (58.3%)	209 (49.6%)	140 (68.0%)	53 (57.0%)	613 (56.7%)
Social deprivation: IRSAD score + mean (SD)	1010.9 (53.6)	1009.2 (51.3)	1009.6 (47.4)	994.6 (62.3)	1008.6 (52.5)
**Medications** ^c^					
Alzheimer's disease	88 (24.3%)	7 (1.7%)	<5	0 (0.0%)	–
Cardiovascular disease	184 (50.8%)	212 (50.4%)	74 (35.9%)	49 (52.7%)	519 (48.0%)
Psychotropic	128 (35.4%)	119 (28.3%)	24 (11.7%)	20 (21.5%)	291 (26.9%)
Antidepressant	142 (39.2%)	169 (40.1%)	32 (15.5%)	13 (14.0%)	356 (32.9%)
**ICD codes** ^d^					
Neurological disorder	90 (24.9%)	128 (30.4%)	11 (5.3%)	5 (5.4%)	234 (21.6%)
Depression	75 (20.7%)	116 (27.6%)	10 (4.9%)	7 (7.5%)	208 (19.2%)
Psychosis	<5	12 (2.9%)	0 (0.0%)	0 (0.0%)	–
Diabetes	70 (19.3%)	73 (17.3%)	16 (7.8%)	19 (20.4%)	178 (16.5%)
Cerebrovascular disease	56 (15.5%)	84 (20.0%)	<5	<5	–
Atrial fibrillation	51 (14.1%)	51 (12.1%)	18 (8.7%)	13 (14.0%)	133 (12.3%)
Chronic kidney disease	40 (11.0%)	33 (7.8%)	7 (3.4%)	6 (6.5%)	86 (7.9%)
**Health‐care interactions**					
*Hospitalization*					
Total emergency presentations, mean (SD)	0.8 (2.1)	0.5 (1.6)	0.4 (1.0)	0.6 (1.4)	0.6 (1.7)
Admission to geriatric ward	126 (34.8%)	120 (28.5%)	15 (7.3%)	11 (11.8%)	272 (25.1%)
Allied health interaction	227 (62.7%)	245 (58.2%)	78 (37.9%)	40 (43.0%)	590 (54.5%)
Behavioral disturbance alarm codes (any)	126 (34.8%)	139 (33.0%)	40 (19.4%)	23 (24.7%)	328 (30.3%)
Aged persons mental health service interaction	27 (7.5%)	31 (7.4%)	0 (0.0%)	0 (0.0%)	58 (5.4%)
Interaction with residential aged care services	130 (35.9%)	66 (15.7%)	<5	<5	–
Readmissions w/in 7 days	39 (10.8%)	46 (10.9%)	13 (6.3%)	<5	‐
*Outpatient clinics*					
Total outpatient appointments, mean (SD)	2.3 (4.9)	3.7 (6.9)	3.2 (5.1)	3.2 (5.2)	3.1 (5.8)
Missed clinic appointments, mean (SD)	0.3 (0.9)	0.4 (1.2)	0.1 (0.4)	0.1 (0.4)	0.3 (1.0)
Neurology outpatient attendance	52 (14.4%)	115 (27.3%)	7 (3.4%)	5 (5.4%)	179 (16.5%)
*Investigations*					
Dementia screening pathology^e^ (any)	108 (29.8%)	121 (28.7%)	42 (20.4%)	20 (21.5%)	291 (26.9%)
Brain imaging	47 (13.0%)	65 (15.4%)	<5	<5	‐

Abbreviations: CDAMS, Cognitive Dementia and Memory Service; ICD, International Classification of Disease; SD, standard deviation.

^a^ TICS‐M = Telephone Interview for Cognitive Status–Modified. Definition of “average” scoring band was based on each individual's age, sex, and educational attainment.^13^

^b^ IRSAD = index of relative socio‐economic advantage and disadvantage, provided at postcode level by the Australian Bureau of statistics.^14^

^c^ Medication names are specified in Table S10 in supporting information.

^d^ Full code sets are available in Table S11 in supporting information.

^e^ Any of: urea and electrolytes, full blood examination, liver function tests, vitamin B12, and thyroid stimulating hormone.

### 3.2 Data reduction (predictor selection)

On average, 4.58 years of data were available for those with dementia. To achieve temporal balance, structured and unstructured data over 4.58 years was included for other groups.

Results from the data reduction survey were as follows: Tier 1, 17 variables; Tier 2, 59 variables; and Tier 3, 22 variables (Table S2). All available Tier 1 variables were included (*n* = 10) and Tier 3 variables excluded. As the final study sample was nearly double the originally anticipated size,^19^ Tier 2 variables were combined in consultation with the expert panel and included as 18 additional predictors, resulting in 28 included predictors (Tables S2, S5 in supporting information).

To guide UMLS concept selection, neurologists identified 163 keywords, geriatricians 457, and medical coders 68 keywords (Box 1). For the full list see https://github.com/NCHADataPlatform/dementia_diagnosis_detection/.

### 3.3 Cohort characteristics

The final structured dataset comprised 44,230 episodes of care, including data from outpatient clinics, inpatient admissions, subacute admissions, and emergency department presentations. Compared to those recruited from the community, individuals attending CDAMS were older; had more appointments and presentations; and higher rates of 7‐day readmission, brain imaging, and screening blood tests (Table 1,.Table S6 in supporting information). Compared to the non‐dementia group, those with dementia were more likely to have dementia ICD codes recorded, to be prescribed anticholinesterase inhibitors or memantine, or have been admitted to geriatric medical ward(s). For unstructured data, the median [interquartile range] document count for a patient attending CDAMS was 63.[1–265] for those with dementia and 50.[5–219] without. For the community‐recruited cohort the median was 32.[16–62] for those scoring average or above average on TICS‐M, and 34.[18–82] for those scoring below average.

### 3.4 Model performance

Performance statistics are reported in Table 2 and variables are described in Table S7 in supporting information.

**TABLE 2:** Model performance.

	Model^a^	AUC^b^	Classification threshold^c^	% correctly classified ^d^ (accuracy)	Sensitivity^e^ (recall)	Specificity^f^	Positive predictive value^g^ (precision)	Negative predictive value^h^
Structured data modeling stream (logistic regression) development, *N* = 1082	Age only	0.67 (0.64–0.70)	0.25	53.1 (0.50–0.56)	85.1 (81.0–88.6)	37.08 (33.5–40.7)	40.5 (37.0–44.1)	83.2 (78.6–87.1)
	Base model	0.81 (0.78–0.83)	0.25	73.38 (0.71–0.76)	72.4 (67.5–76.9)	73.89 (70.5–77.1)	58.22 (53.5–62.8)	84.18 (81.1–86.9)
	Full model	0.83 (0.81–0.86)	0.25	74.50 (0.71–0.77)	80.4 (75.9–84.4)	71.5 (68.1–74.8)	58.7 (54.2–63.0)	87.9 (85.0–90.4)
	LASSO penalized	0.84 (0.81–0.86)	0.25	74.9 (0.72–0.77)	80.7 (76.2–84.6)	71.9 (68.4–75.1)	59.11 (54.5–63.4)	88.10 (85.2–90.6)
Structured data modeling: performance among those with unstructured (text) data *N* = 860	Age only	0.68 (0.64–0.72)	0.25	52.0 (49.0–55.0)	85.8 (80.8–89.8)	37.9 (34.0–41.9)	36.5 (32.7–40.5)	86.5 (81.8–90.3)
	Base model	0.86 (0.83–0.89)	0.25	77.0 (74.0–80.0)	82.6 (77.4–87.1)	74.6 (71.0–78.0)	57.6 (52.3–62.7)	91.1 (88.3–93.5)
	Full model	0.86 (0.84–0.89)	0.25	0.77 (74.4–80.0)	83.0 (77.8–87.4)	75.0 (71.3–78.4)	58.0 (52.7–63.1)	91.4 (88.5–93.7)
	LASSO penalized	0.86 (0.84–0.89)	0.25	0.77 (0.74–0.80)	82.6 (77.4–87.1)	75.0 (71.3–78.4)	57.9 (52.6–63.0)	91.2 (88.3–93.5)
NLP modeling stream development, *N* = 860	Keyword	0.520	–	37.39	94.47	9.62	33.71	78.12
	Logistic regression	0.792 (0.754–0.830)	0.35	81.18 (77.75–84.60)	73.23 (66.99–79.48)	85.23 (81.46–89.00)	71.19 (64.07–78.31)	86.44 (82.95–89.93)
	Naïve Bayes	0.665 (0.629‐ 0.701)	0.25	68.92 (65.65–72.18)	59.96 (53.94–65.98)	73.17 (69.40–76.94)	51.48 (45.87–57.09)	79.38 (75.89–82.88)
	Support vector machine	0.804 (0.772–0.836)	0.30	82.63 (79.63–83.69)	74.03 (68.60–79.46)	86.92 (83.14–90.69)	73.56 (66.54–80.58)	87.20 (84.08–90.33)
	Adaboost	0.784 (0.753–0.814)	0.50	82.17 (79.58–81.42)	67.14 (61.19–73.08)	89.66 (86.45–92.86)	76.37 (69.68–83.06)	84.65 (81.50–87.79)
	Random forest	0.794 (0.762–0.826)	0.40	83.01 (80.32–85.70)	68.67 (62.44–74.90)	90.23 (86.99–93.47)	78.14 (71.78–84.49)	85.20 (81.96–88.23)
Combined stream (logistic regression) *N* = 860	LASSO + NLP	0.94 (0.93–0.96)	0.30	86.86 (0.84–0.89)	92.8 (89.7–95.3)	72.2 (68.8–75.5)	76.9 (67.9–77.9)	95.2 (93.1–96.9)

*Note*: All results in parentheses are 95% confidence intervals.

Abbreviations: AUC, area under the curve; ICD, International Classification of Disease; LASSO, least absolute shrinkage and selection operator; NLP, natural language processing.

^a^ “Age only” model included age as a predictor; “base” model included age, sex, social deprivation score, presence of dementia ICD‐10 Code. Full model included 28 predictors assembled by the clinical expert panel (see Table S9 in supporting information for descriptions of all variables).

^b^ Area under the receiver operator characteristics curve. For unstructured data models, estimated via cross‐validation (100 replications). For structured data models, the AUC was optimism corrected using 200 bootstrap repetitions.

^c^ The classification threshold was tuned for each individual model, by selecting a value that appeared to jointly maximize accuracy, sensitivity/recall, and specificity, subject to our preference for sensitivity above specificity.

^d^ Proportional correctly classified/accuracy: overall proportion of patients correctly classified using this model (at the specified threshold).

^e^ Sensitivity: given a patient has a dementia diagnosis, the probability they are correctly classified as a case by this model (at the specified threshold).

^f^ Specificity: given a patient does not have a dementia diagnosis, the probability they are correctly classified as a non‐case by this model (at the specified threshold).

^g^ Positive predictive value/precision: Given the model has classified this patient as a case (positive prediction), what is the probability that they *do* have a dementia diagnosis?

^h^ Negative predictive value: Given the model has classified this patient as a non‐case (negative prediction), what is the probability that they *do not* have a dementia diagnosis?

#### 3.4.1 Structured modeling

A classification threshold of 25% was judged optimum for all models. The age‐only model performed similarly to a random classifier (accuracy: 53.1%.[0.50–0.56], AUC.0.67.[95%.confidence interval (CI).0.64– 0.70]). The base model demonstrated substantial improvement over the age‐only model (accuracy: 73.4%.[0.71–0.76], AUC.0.81.[0.78–0.83]). The full model represented a statistically significant improvement over the base model (AUC [full] = 0.85, AUC [base] = 0.81, test of AUC equivalence chi^2 ^= 18.8, *P* < 0.0001).

Sixteen predictors were retained after LASSO penalization (Table S5) and performed comparably to the 28‐predictor model (Table 2). Regularized coefficients for retained variables are presented in Table S8 in supporting information. Ultimately, 60% of included Tier 1 (6/10) and Tier 2 variables (11/18) were retained by LASSO. Performance of all structured models was substantively unchanged when restricted to the 860 patients with text available (Table 2). In sensitivity analyses, the same variables were retained by LASSO even when those scoring below average on TICS‐M were excluded from the non‐dementia group (Table S9 in supporting information).

#### 3.4.2 Unstructured/NLP modeling stream

After sensitivity optimization, classification thresholds for NLP models ranged from 35% to 50%. Keyword search was inferior to a random classifier (accuracy: 35%). Statistical and machine‐learning classifiers demonstrated substantial improvement. The best performing classifiers were random forest (accuracy 83%.[80.3–85.7]), SVM (accuracy 82%.[79.6–83.7]), logistic regression (accuracy 81%.[77.8–84.6]), and adaptive boosting (accuracy 82%.[79.6–83.7]). Among these, none demonstrated statistical superiority (all *P* > 0.10 for AUC equivalence). The most informative concepts for classification using random forest, based on permutation test (*P* < 0.01), were “dementia,” “care of aged facility,” “memory,” “confusion,” and “disorientation.” For details of NLP models and concepts see http://github.com/NCHADataPlatform/dementia_diagnosis_detection.

#### 3.4.3 Combined model

**FIGURE 3:**
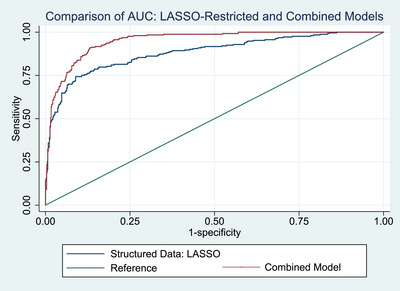
Comparison of area under the receiver operator characteristics curves. “Structured data: LASSO” (*n* = 1082) refers to the result of subjecting the full model (28 predictors) to LASSO penalization, after which 16 variables were retained. The “Combined model” (nested, *n* = 860) drew upon structured and unstructured insights, by incorporating the NLP‐derived prediction into the Structured, LASSO‐penalized model. AUC, area under the curve; LASSO,  least absolute shrinkage and selection operator; NLP, natural language processing.

Given equivalent performance of NLP classifiers, the patient‐level prediction from the random forest was passed to the structured model. Random forest was selected as the ensemble approach is generally more stable than predictions from single‐structure models, and less vulnerable to overfitting than AdaBoost or SVM. The resulting combined model substantially improved performance compared to the LASSO‐penalized structured model (Figure 3, AUC.0.94.[0.93–0.96], accuracy 86%.[0.84–0.89], test of AUC equivalence chi^2 ^= 40.22,.*P* < 0.0001).

## 4 DISCUSSION

We present new evidence demonstrating the value of combining structured (using traditional biostatistical approaches) and unstructured[Table alz70132-tbl-0003] (using NLP/data science approaches) algorithm development streams to improve capture and recognition of people with diagnosed dementia from EHRs. Our combined algorithm demonstrated high classification accuracy for people with and without dementia, reflected in high sensitivity and specificity. Final models from both development streams showed good balance between sensitivity and specificity, with slightly higher positive predictive value (PPV) for NLP models. Results suggest that EHRs contain routinely collected, sensitive indicators that may contribute to substantially improved accuracy in population‐wide capture of dementia, over and above routinely reported administrative data.

Embedding text‐derived probability scores within expert‐led, structured algorithms appears to meaningfully enhance predictive power. While prior studies have incorporated unstructured data elements into predictive tools, methodological issues with these studies include small sample sizes,^5^, ^25^ absence of substantial expert involvement,^5^, ^7^, ^25^ and unclear case definition or reliance on ICD codes for ascertainment.^5^, ^7^, ^8^, ^26^ The combination of predictors derived from structured and unstructured streams demonstrated clear superiority; however, our results also suggest comparable performance between structured and unstructured algorithms developed with expert guidance (final structured models demonstrating similar AUC but slightly lower accuracy than NLP classifiers). After validation, these models might be usefully applied in contexts in which only structured or unstructured data are available.

There is significant heterogeneity in the current literature in terms of precise model aims (predicting future dementia risk, detecting unrecognized/undiagnosed dementia, versus detecting patients who have already been diagnosed), data sources (primary care, insurance databases, health service, and Veterans Affairs), and performance metric selection (sensitivity/specificity, PPV, F1, AUC). This makes a straightforward comparison of studies extremely difficult.

Our sensitivity/PPV (92.8/76.9) is at the upper end of the ranges (21–86 and 33–100) reported by a systematic review^27^ examining dementia coding in routinely collected data. This review identified primary care data as being able to identify cases with a high PPV, while US insurance data produced the highest sensitivity. This review also noted the heterogeneity in data sources and study questions.

More recent models trained with EHR data for dementia detection are more likely to use unstructured text data. Table 3 summarizes the characteristics and discriminative performance of recent studies that use unstructured text data,^5^, ^7^, ^8^, ^25^, ^26^, ^28^, ^29^, ^30^ except for the eRADAR model by Barnes et al.,^6^ which only uses structured data. It is evident that our discriminative performance (AUC 0.94) and accuracy (86.9%) are superior compared to similar studies (AUC/C statistic 0.62–0.91, accuracy 73.5%–77.4%). Classification measures such as model sensitivity, specificity, PPV, and negative predictive value are harder to compare between these studies as they either use varying probability thresholds or do not report the thresholds used to generate such measures.

**TABLE 3 alz70132-tbl-0003:** Performance of selected dementia algorithms using EHR data.

Study	Country	Data setting and source	Type of data	Aim	Discriminative performance	Accuracy	Other performance metrics
Collyer, 2025	Australia	Tertiary care EHR	Structured and unstructured	To detect diagnosed dementia	AUROC 0.94	86.9%	N/A
Barnes, 2020	USA	Insurance database with integrated primary/secondary/tertiary EHR (Kaiser Permanente)	Structured	To detect unrecognized dementia	C‐statistic 0.81	Not reported	N/A
Maclagan, 2023	Canada	Primary care EHR (EMRPC database)	Unstructured	To detect unrecognized dementia	Not reported	Not reported	F1 score 77.2%
McCoy, 2020	USA	Tertiary care EHR (discharge documents)	Unstructured	To stratify future dementia risk	C‐index 0.62	Not reported	HR 1.5 in pooled analysis of 2 hospitals
Amra, 2017	USA	ICU EHR	Unstructured	To identify cognitive impairment and dementia	Not reported	Not reported	Sensitivity 96%, specificity 100% in validation cohort
Shao, 2019	USA	Veterans EHR	Structured and unstructured	To detect unrecognized dementia	AUROC 0.91	Not reported	N/A
Boustani, 2020	USA	Integrated primary, secondary, and tertiary care EHR (Indiana Network for Patient Care)	Structured and unstructured	Future dementia risk in 1, 3, and 5 years	AUROC 0.80 at 1 year, 0.75 at 3 years, and 0.70 at 5 years	Not reported	N/A
Reuben, 2017	USA	UCLA EHR	Structured and unstructured	To detect unrecognized dementia	Not reported	Not reported	PPV 87%, sensitivity 34%
Ben Miled, 2020	USA	Integrated primary, secondary, and tertiary care EHR (Indiana Network for Patient Care)	Structured and unstructured	To predict future dementia risk in 1 and 3 years	Not reported	77.4% at 1 year, 73.5% at 3 years (Cases and controls balanced 1:1 in training set)	N/A
Zolnoori, 2025	USA	Transcriptions of recordings of nurse–patient interactions during home care + EHR.	Structured and unstructured	Detection of early mild cognitive impairment	90.23	Not reported	F1 score 88.89

*Note*: Data types, settings, study aims and performance metrics for recent studies using machine learning and EHR data in dementia contexts.

Abbreviations: AUROC, area under the receiver operating characteristic curve; EHR, electronic health record; EMRPC, Electronic Medical Record Primary Care; HR, hazard ratio; ICU, intensive care unit; PPV, positive predictive value; UCLA, University of California Los Angeles.

Evidence for superiority of a combined development approach carries implications not only for the epidemiology of dementia, but perhaps also for efficient identification of people with high probability of undiagnosed dementia, suitable for clinical care decision pathways, and clinical measures important in estimating dementia severity.^31^ Given that clinical recognition of people diagnosed with dementia presenting to hospitals is poor,^32^ placing them at risk of delirium and significant complications, our method provides a strategy for capturing and combining clues in written text (e.g., descriptions of confusion, forgetfulness) and structured data that are often ignored (e.g., behavioral agitation alerts), to flag such people for appropriate diagnostic and clinical care.

The validity of our findings is enhanced by use of gold‐standard, specialist clinical impressions against Diagnostic and Statistical Manual of Mental Disorders criteria in case ascertainment, not possible in prior studies aiming to detect incidents or early dementia from EHRs. Previous authors generally relied upon administrative diagnostic codes,^8^, ^26^, ^29^ known to poorly capture diagnosed dementia in Australian contexts^33^ and highly variable in global contexts.^34^ Our incorporation of these codes as predictors (rather than outcomes) represents a major advantage over prior algorithms for detecting dementia, and likely explains the superior performance of our structured models, compared to other studies. Additionally, while prior studies of dementia have engaged experts in keyword or concept selection^5^, ^8^, ^26^ ours is the first study to subject a dual‐stream development process to oversight from an international, clinically diverse panel, and to combine this with gold‐standard case ascertainment. The panel's efforts to systematically filter and prioritize the vast quantity of EHR data available reduced data processing requirements, and provides greater confidence in stability of model performance.^15^ Our approach provides a framework for efficient use of machine learning techniques for capture of dementia (and other conditions) from large volumes of available unstructured and structured medical data.

Our study also had the advantage of access to a data platform derived directly from EHR systems, optimized for research.^12^ Our training data were generated from comprehensive structured and unstructured datasets from real (production) EHR systems. For structured data, in addition to ICD codes for dementia, we were able to include variables reflecting demographics, socioeconomic status, pharmacological therapy, emergency and clinic health use, and in‐hospital events (such as agitated behavior) not currently captured within administrative datasets. For unstructured data, a significant advantage was that we accessed hospital records collected across diverse clinical settings, rather than relying upon data from a single ward,^5^ or upon models pre‐trained using a single document type.

Limitations to our study include a relatively small, ethnically homogeneous sample,^13^ which limited the number of candidate predictors included in structured models, and precluded reserving a portion of the data for validation.^15^, ^20^ However, expert involvement in variable selection and optimism‐correction of performance statistics reduces risk of overfitting.^16^ Final models require validation using data from different individuals, or in different settings. Importantly, however, our presentation of performance benchmark models, and results from independent modeling streams, provides meaningful context for interpreting algorithmic performance. Finally, we did not adopt a meta‐classifier approach or attempt to implement large language models, due to limited training data. For cybersecurity and confidentiality reasons, such models cannot be feasibly applied to real Australian EHR datasets held by public providers.

In conclusion, as routinely collected data provide the most cost‐effective method for long‐term monitoring of dementia prevalence,^4^ and indeed other diseases, our novel dual‐stream development approach provides an opportunity for enhanced, efficient, and sustainable population health monitoring. We present novel evidence that the combination of insights from structured and unstructured development streams provides a significant opportunity for capture of diagnostic status from EHR data, with benefits potentially arising for dementia epidemiology and beyond.

## CONFLICT OF INTEREST STATEMENT

The authors have no conflicts of interest pertaining to the content of this manuscript.

Author disclosures are available in the supporting information.

## ETHICS STATEMENT

The project was approved by the Peninsula Health Human Research Ethics Committee (HREC/58604/PH‐2019‐194324) and Monash University Human Research Ethics Committee (MUHREC/22080).

## CONSENT STATEMENT

All participants recruited from the community provided informed consent prior to telephone interview. Consent was not necessary for those assessed via CDAMS, as the NCHA data platform holds a waiver of consent for extraction of routinely collected data from relevant clinical systems. These patients were not contacted for any reason regarding this study.

## Supporting information



Supporting Information

Supporting Information

Supporting Information
