# Supplementary material for: Dual‐stream algorithms for dementia detection: Harnessing structured and unstructured electronic health record data, a novel approach to prevalence estimation
Source: Alzheimers Dement. 2025 May 5;21(5):e70132. doi: 10.1002/alz.70132 (PMC12053150; doi:10.1002/alz.70132)

**Supplementary Table 1** – Data Sources
The six sources detailed below reflect the location of relevant Data within the NCHA Data platform. (The Platform extracts, cleans and integrates data from 8 clinical systems relevant to this project.)

| **Dataset** | **Description** |
| --- | --- |
| Demographics | Patient demographic information (e.g. age, gender and postcode) |
| Memory Clinic  Used only to identify dementia cases. Not used for model covariates. | This multidisciplinary memory clinic operates on-site at Peninsula Health and is funded by the state government. The clinic specialises in the diagnosis of dementia for community-dwelling people.  Data obtained included referral dates, attendance dates, imaging dates and diagnosis dates. We relied primarily on memory clinic data for our case ascertainment. We confirmed all diagnosis dates by scrutinising correspondence between the diagnosing specialist and individuals’ usual primary care provider. |
| Community Health | ‘Community health’ refers to a set of universally accessible services available to people not admitted to hospital, including a number of targeted services for vulnerable population groups. Examples include smoking cessation support, nutrition, allied health services, dentistry and sexual health.   Community Health works alongside general practice and privately funded services to make up the primary health sector in Victoria. Available data included information regarding the assessment and provision of support for home and community care. |
| Inpatient Admissions | Inpatient admissions & emergency department (ED) presentations datasets included all planned (elective) and unplanned admissions and ED presentations to Peninsula Health from two acute care and two sub acute care (rehabilitation) hospitals.  The data includes information on patients’ admission and discharge dates, length of stay, diagnoses, surgical procedures, medications dispensed, and imaging. |
| ED Presentations |  |
| Outpatient | Outpatient care refers to medical care provided to patients in settings outside of the hospital, such as in a clinic or a doctor's office (e.g. neurologist). Data included information regarding appointment types, physician specialty, appointment lengths/costs, visit dates and times, laboratory tests, services provided, medication lists, and patient outcomes. |

##

## **Supplementary Table 2** – Candidate Predictors, Structured Data

| **Candidate Predictor** | **Tier** | **Included in Full Model** |
| --- | --- | --- |
| Age | 1 | Yes |
| Dementia ICD Code | 1 | Yes |
| Admission to Residential Aged Care | 1 | Yes |
| Attended CDAMS/cognitive clinic | 1 | No, this was an inclusion/exclusion criteria |
| Attended neuropsychology | 1 | No, data were unavailable |
| Admitted to geriatric ward psychogeriatric unit | 1 | Yes |
| Recorded Neurology Outpatients Appointment | 1 | Yes |
| Recorded use of Aged Persons Mental Health service | 1 | Yes |
| Code grey/s recorded | 1 | Yes |
| Mini Mental State Examination (MMSE) | 1 | No, data were unavailable |
| Montreal Cognitive Assessment (MoCA) | 1 | No, data were unavailable |
| 4AT - Rapid Clinical Test for Delirium | 1 | No, data were unavailable |
| Neuropsychiatric Inventory | 1 | No, data were unavailable |
| History of Cerebrovascular disease | 1 | Yes |
| Medication nonadherence | 1 | No, data were unavailable |
| Cholinesterase inhibitors | 1 | Yes |
| CT Brain | 1 | Yes, as combined brain imaging indicator |
| MRI brain | 1 |  |
| SPECT | 1 |  |
| Sex | 2 | Yes |
| Obese, BMI ≥ 30 | 2 | No, data were unavailable |
| Systolic blood pressure | 2 | No, data were unavailable, but use of hypertension medications was included |
| High BP, ≥ 140 SBP or 90 DBP | 2 |  |
| Number of Emergency Department (ED) visits | 2 | Yes |
| Potentially avoidable hospitalisations | 2 | No, data were unavailable |
| Recurrent admissions/ readmitted within a week | 2 | Yes |
| Awaiting admission to other healthcare facility | 2 | No, data were unavailable |
| Care involving use of rehabilitation procedure | 2 | No, data were unavailable |
| Clinic no shows | 2 | Yes |
| Number of Outpatient visits | 2 | Yes |
| Number of Home health visits | 2 | No, data were too sparse |
| Recorded use of Falls and Balance Clinic | 2 | No, data were too sparse |
| Recorded use of Allied health services | 2 | Yes |
| Referred to Occupation Therapy (OT)/ Social Work (SW) | 2 | No, data were unavailable |
| Recorded use of Carer services | 2 | No, data were too sparse |
| iPm “advised to contact Next Of Kin first” flag | 2 | No, data were too sparse |
| Dementia severity (Global Dementia Rating scale [GDR]) | 2 | No, data were unavailable |
| Telephone Interview for Cognitive Status-modified (TICS-M) Raw Score | 2 | No, data were unavailable |
| TICS-M Classification (e.g. mildly impaired, low average, average, superior) | 2 | No, data were unavailable |
| Congestive Heart Failure | 2 | Yes, combined CVD variable |
| Diabetes, any | 2 | Yes, combined diabetes indicator |
| Diabetes, complex | 2 |  |
| Hypothyroidism | 2 | No, data too sparse |
| Renal failure | 2 | Yes, combined CKD indicator |
| Fluid and electrolyte disorders | 2 |  |
| Depression | 2 | Yes |
| Traumatic brain injury | 2 | No, data too sparse |
| Tobacco use disorder | 2 | No, data were unavailable |
| Stroke | 2 | Yes |
| Hypertension | 2 | Yes |
| Atrial fibrillation | 2 | Yes |
| Gait Abnormality | 2 | No, data were unavailable |
| Recorded use of drug/s related to sleeping, anxiety, psychotics and depression | 2 | Yes, combined antidepressant usage indicator |
| Nontricyclic antidepressants | 2 |  |
| Sedative/hypnotic | 2 |  |
| Number of medications | 2 | No |
| Start date of psychotropics | 2 | No, data too sparse |
| Stop date of psychotropics | 2 | No, data too sparse |
| Overdose | 2 | No, data too sparse |
| Drug toxicity | 2 | No, data were unavailable |
| Opioid | 2 | No |
| Benzodiazepines | 2 | Yes, combined antidepressant indicator |
| Psychoactive drugs | 2 | Yes |
| UEC | 2 | Yes, combined screening pathology indicator |
| FBE | 2 |  |
| LFT | 2 |  |
| B12 | 2 |  |
| TSH | 2 |  |
| Urine MCS | 2 |  |
| Syphilis (RPR) | 2 |  |
| VDRL | 2 |  |
| HIV | 2 | No, data too sparse |
| Anti-neuronal antibodies, 14-3-3, RTQuick | 2 | No, data too sparse |
| FDG PET | 2 | Yes, combined brain imaging indicator |
| CSF | 2 | No, data too sparse |
| Recorded use of Respite services | 2 | No, data unavailable |
| Psychosis | 2 | Yes |
| Marital status | 3 | No |
| Language, culturally and linguistically diverse (CALD) | 3 | No |
| Aboriginal and/or Torres Strait Islander | 3 | No |
| Living arrangement | 3 | No |
| Postcode | 3 | No |
| Height | 3 | No |
| Weight | 3 | No |
| BMI | 3 | No |
| Underweight, BMI < 15.5 | 3 | No |
| Diastolic blood pressure | 3 | No |
| Over what period of time are health utilisation data (see below) relevant? | 3 | No |
| Number of Physical therapy visits | 3 | No |
| Number of Speech, language and learning visits | 3 | No |
| Over what period of time are service data (see below) relevant? | 3 | No |
| Charlson Comorbidity Index | 3 | No |
| Elixhauser Index | 3 | No |
| Chronic pulmonary disease | 3 | No |
| Lymphoma | 3 | No |
| Solid tumor without metastases | 3 | No |
| Rheumatoid arthritis | 3 | No |
| Blood loss anaemia | 3 | No |
| Vitamin D | 3 | No |

## **Supplementary Table 3: Study corpus characteristics**

| Cohort (Patients who have unstructured data) | Confirmed Dementia: CDAMS (n=253) | Confirmed non Dementia: CDAMS (n=339) | >= Average  on TICS-M (n=181) | < Average  on TICS M (n=87) |
| --- | --- | --- | --- | --- |
| Average count of clinical documents (per patient) | 221.70 | 218.76 | 60.39 | 62.01 |
| Average document length (in tokens*, per patient) | 869.72 | 929.34 | 807.38 | 843.34 |

## **Supplementary Table 4** – Review of ambiguous diagnostic terminology

| *Available Text* | *Decision* | *Notes from discussion and review of records* |
| --- | --- | --- |
| OCT | Not Confirmed Dementia | Confirmed Typo. This is OCI: "Other Cognitive Impairment" |
| AD | Confirmed Dementia |  |
| AD / VASC | Confirmed Dementia |  |
| AD / VD | Confirmed Dementia |  |
| AD/VASC | Confirmed Dementia |  |
| ALC MCI DEP | Not Confirmed Dementia | Notes stated: Alcohol, MCI & depression |
| ALZ | Confirmed Dementia |  |
| ALZ / VASC | Confirmed Dementia |  |
| Atypical AD | Confirmed Dementia |  |
| DEM | Confirmed Dementia |  |
| DEM LB | Confirmed Dementia |  |
| DLB/VD/AD | Confirmed Dementia |  |
| EARLY AD | Confirmed Dementia |  |
| FRONT AD | Confirmed Dementia |  |
| FTD | Confirmed Dementia |  |
| MCI | Not Confirmed Dementia |  |
| MCI AD | Confirmed Dementia | Notes stated "mild cognitive impairment with an Alzheimer's component likely. This was consistent with history as well as a previous MRI scan which demonstrated hippocampal atrophy." |
| MIX VAS AD | Confirmed Dementia |  |
| MIXED | Confirmed Dementia |  |
| MIXED | Confirmed Dementia |  |
| N/A | Not Confirmed Dementia |  |
| NAD | Not Confirmed Dementia |  |
| NO | Not Confirmed Dementia |  |
| NO DEM | Not Confirmed Dementia |  |
| NO DIAG | Not Confirmed Dementia |  |
| NOD | Not Confirmed Dementia |  |
| NONE | Not Confirmed Dementia |  |
| OCI | Not Confirmed Dementia | Other Cognitive Impairment |
| OTHER | Not Confirmed Dementia |  |
| PARK DEM | Confirmed Dementia | Notes state Parkinsons dementia |
| PD | Not Confirmed Dementia | Listed as dx for 4 patients: Pt.1: MCI due to parkinsons disease; Pt.2: Parkinson's disease with dementia.Pt.3: MCI, background of Parkinson disease., Pt.4: Parkinson's disease |
| PD / AD | Confirmed Dementia |  |
| PPA | Confirmed Dementia | Primary Progressive Aphasia |
| STROKE | Not Confirmed Dementia |  |
| VASC D | Confirmed Dementia | Vascular Dementia |
| VCI | Not Confirmed Dementia | Vascular Cognitive impairment |
| VCI / DEP | Not Confirmed Dementia | as above with depression |
| VD | Confirmed Dementia | Vascular Dementia |

## Supplementary Table 5 - Structured Model Specification

As the final sample size was almost double the projected sample size, and observed R^2^ substantially higher than anticipated, we allowed inclusion of 11 additional candidate predictors in the Full model.

| Model | Specification |
| --- | --- |
| Age Only | Logit(p[Dementia]) = -0.737 + 0.074[Age] |
| Base | Logit(p[Dementia]) = -1.580 + 0.056[Age] + 0.315[FemaleSex] +0.002[IRSAD] + 2.565[DementiaICDCode] |
| Full Model | Logit(p[Dementia]) = -1.008 + 0.063[Age] + 0.270[FemaleSex] + 0.001[IRSAD] + 2.967[DementiaICDCode] – 0.345[NeurologistOutpatients] + 0.091[Readmitted7Days] + 0.661[AgedCare] – 0.082[GeriatricCare] + 0.0193[AgedMHService] – 0.797[CodeGreys] – 0.022[AlliedHealth] – 0.167[BrainImaging] + 2.436[ADMedications] – 0.554[CVDMedication] + 0.167[Psychotropics] – 0.136[Antidepressants] + 0.178[CV_Dis] – 0.596[NeurologicalDisorder] – 0.889[Psychosis] – 0.100[DiabetesICD] - 0.284[DepressionICD] + 0.197[AtrialFibrilation] -0.488[ICD_CVD] – 0.125[ICD_CKD] – 0.023[LaboratoryTests] – 0.078[EmergencyAttendance] – 0.024[OutpatientsAttendance] – 0.053[ClinicNoShows] |
| LASSO-Restricted | Logit(p[Dementia]) = -1.010 + 0.063[Age] + 0.0261[FemaleSex] + 0.001[IRSAD] + 2.938[DementiaICDCode] – 0.313[NeurologistOutpatients] + 0.683[AgedCare] + 0.797[CodeGreys] + 2.406[ADMedications] – 0.574[CVDMedication] + 0.178[CV_Dis] – 0.609[NeurologicalDisorder] – 0.840[Psychosis] - 0.318[DepressionICD] – 0.430[ICD_CVD] – 0.070[EmergencyAttendance] – 0.027[OutpatientsAttendance] |

##

## Supplementary Table 6 - Characteristics of patients with sufficient unstructured data (N=860)

|  | Diagnosed Dementia  (n=253) | Not Diagnosed Dementia (n=607) | | | Total  N=860 |
| --- | --- | --- | --- | --- | --- |
|  | Confirmed Dementia: CDAMS | Confirmed Non-Dementia: CDAMS | >= Average  on TICS-M | < Average  on TICS M |  |
|  | N=253 | N=339 | N=181 | N=87 | N=860 |
| Age, mean (SD) | 80.0 (7.1) | 77.4 (8.1) | 71.7 (6.2) | 73.7 (7.7) | 76.6 (8.0) |
| Female Sex | 138 (54.5%) | 170 (50.1%) | 119 (65.7%) | 49 (56.3%) | 476 (55.3%) |
| IRSAD Score (Social Deprivation), mean (SD) | 1009.4 (54.5) | 1006.2 (50.8) | 1006.6 (46.9) | 995.2 (63.9) | 1006.1 (52.7) |
| **Medications** |  | | | | |
| Alzheimer's Disease Medications | 82 (32.4%) | 7 (2.1%) | <5 (X%) | 0 (0.0%) | - |
| CVD Medications | 164 (64.8%) | 195 (57.5%) | 70 (38.7%) | 47 (54.0%) | 476 (55.3%) |
| Psychotropic Medications | 116 (45.8%) | 110 (32.4%) | 23 (12.7%) | 19 (21.8%) | 268 (31.2%) |
| Antidepressant Medications | 131 (51.8%) | 156 (46.0%) | 29 (16.0%) | 13 (14.9%) | 329 (38.3%) |
| **ICD Codes** |  | | | | |
| Neurological Disorder ICD Code | 80 (31.6%) | 118 (34.8%) | 11 (6.1%) | 5 (5.7%) | 214 (24.9%) |
| Depression ICD Code | 68 (26.9%) | 110 (32.4%) | 10 (5.5%) | 7 (8.0%) | 195 (22.7%) |
| Diabetes ICD Code | 61 (24.1%) | 68 (20.1%) | 15 (8.3%) | 18 (20.7%) | 162 (18.8%) |
| CV ICD Code | 50 (19.8%) | 75 (22.1%) | <5 (X%) | <5 (X%) | - |
| Atrial Fibrillation ICD Code | 45 (17.8%) | 46 (13.6%) | 18 (9.9%) | 11 (12.6%) | 120 (14.0%) |
| Chronic Kidney Disease ICD Code | 37 (14.6%) | 31 (9.1%) | 6 (3.3%) | 6 (6.9%) | 80 (9.3%) |
| Psychosis ICD Code | <5 (0.8%) | 11 (3.2%) | 0 (0.0%) | 0 (0.0%) | - |
| **Healthcare Utilisation / Interactions** |  | | | | |
| Allied Health use | 182 (71.9%) | 221 (65.2%) | 68 (37.6%) | 38 (43.7%) | 509 (59.2%) |
| Neurologist outpatient attendance | 44 (17.4%) | 106 (31.3%) | 6 (3.3%) | 5 (5.7%) | 161 (18.7%) |
| Code Greys (any) | 126 (49.8%) | 138 (40.7%) | 40 (22.1%) | 23 (26.4%) | 327 (38.0%) |
| Missed Appointments (any), mean (SD) | 0.3 (1.0) | 0.5 (1.4) | 0.1 (0.4) | 0.1 (0.4) | 0.3 (1.1) |
| Interaction w. Residential Aged Care Services | 116 (45.8%) | 60 (17.7%) | <5 (X%) | <5 (X%) | - |
| Admitted to Geriatric Ward | 113 (44.7%) | 111 (32.7%) | 12 (6.6%) | 11 (12.6%) | 247 (28.7%) |
| Accessed aged persons mental health service | 26 (10.3%) | 29 (8.6%) | 0 (0.0%) | 0 (0.0%) | 55 (6.4%) |
| Dementia Screening Pathology (Any) | 96 (37.9%) | 110 (32.4%) | 39 (21.5%) | 19 (21.8%) | 264 (30.7%) |
| Brain Imaging | 43 (17.0%) | 60 (17.7%) | <5 (X%) | <5 (X%) | - |
| Total Outpatient Appointments, mean (SD) | 2.9 (5.4) | 4.4 (7.4) | 3.4 (5.3) | 3.2 (5.1) | 3.6 (6.3) |
| Total ED Presentations, mean (SD) | 1.1 (2.4) | 0.6 (1.7) | 0.5 (1.0) | 0.6 (1.4) | 0.7 (1.8) |
| Readmissions w/in 7 days | 37 (14.6%) | 43 (12.7%) | 10 (5.5%) | <5 (X%) | - |

## Supplementary Table 7: Structured modelling stream, Included predictors

Data Sources are colour-coded and described in Supplementary Table 1

| **Variables** | **Possible Values** | **Data Source, description & applicable time periods** |
| --- | --- | --- |
| **Age** on index date (Jan 1, 2015) | Continuous, non-integer (≥60 years) | **Demographics** Age of patient  **Time frame:**   - **Dementia Cohort** - Age at diagnosis - **Non-Dementia Cohort** - Age at commencement of cognitive screening |
| **Female** (Gender) | 0 = no 1 = yes | **Demographics** Patient identified as female.  (Note: In our dataset, all individuals identified as either Male or Female gender).  **Non Time-Varying in extracted EHR.** |
| **Index of Relative Socio-economic Advantage and Disadvantage** (**IRSAD**) score | Score | **Demographics** This score is a relative measure of postcode area-level socioeconomic position and is intended for enabling comparison between areas. The score is derived from 17 indicators of disadvantage and 8 for advantage. A low score indicates relatively greater disadvantage and a lack of advantage, while a high score indicates a relative lack of disadvantage and greater advantage. The scores do not have intrinsic meaning or are a reflection of outcomes for individuals in the region.  **Non Time-Varying in extracted EHR.** |
| **Number of ED** **visits** | Count | **ED Presentations*** Total number of visits to the emergency department (annualised). |
| **Admitted to geriatric ward, locked ward or psychogeriatric unit** | 0 = no 1 = yes | **Inpatient Admissions*** Denotes whether or not the patient was admitted to a geriatric ward, locked ward or received care from a psychogeriatric unit. |
| **Behavioural Disturbance Codes** (i.e. Code Grey) | 0 = no 1 = yes | **Inpatient Admissions*** Denotes whether or not a code grey was recorded for a patient. It is defined as “an organisation-level response to actual or potential violent, aggressive, abusive or threatening behaviour, exhibited by patients or visitors, towards others or themselves, which creates a risk to health and safety” [Code Grey and Code Black (health.vic.gov.au)](https://www.health.vic.gov.au/worker-health-wellbeing/code-grey-and-code-black) |
| **Readmitted within a week** | 0 = no 1 = yes | **Inpatient Admissions*** Patient was readmitted to hospital within 7 days. Admissions related to dialysis were excluded. |
| **Number of clinic no-shows** | Count | **Outpatient*** Number of ‘no shows’ per year (i.e. annualised failures per year to attend scheduled appointments at outpatient clinics). |
| **Number of outpatient visits** | Count | **Outpatient*** Total number of visits to outpatient clinics |
| **Atrial fibrillation** (ICD-10) | 0 = no 1 = yes | **Inpatient Admissions + ED Presentations*** Denotes whether or not the patient had a primary or secondary diagnosis related to atrial fibrillation and were identified using ICD-10 codes. |
| **Cerebrovascular disease** (ICD-10) | 0 = no 1 = yes | **Inpatient Admissions + ED Presentations*** Denotes whether or not the patient had a primary or secondary diagnosis related to cerebrovascular disease or traumatic brain injury and were identified using ICD-10 codes. |
| **Chronic renal failure** (ICD-10) | 0 = no 1 = yes | **Inpatient Admissions + ED Presentations*** Denotes whether or not the patient had a primary or secondary diagnosis related to chronic renal failure or dialysis and were identified using ICD-10 codes. |
| **CVD** (ICD-10) | 0 = no 1 = yes | **Inpatient Admissions + ED Presentations*** Denotes whether or not the patient had a primary or secondary diagnosis related to cardiovascular disease and were identified using ICD-10 codes. |
| **Dementia** (ICD-10) | 0 = no 1 = yes | **Inpatient Admissions + ED Presentations*** Denotes whether or not the patient had a primary or secondary diagnosis related to dementia and were identified using ICD-10 codes. |
| **Depression** (ICD-10) | 0 = no 1 = yes | **Inpatient Admissions + ED Presentations*** Denotes whether or not the patient had a primary or secondary diagnosis related to depression and were identified using ICD-10 codes. |
| **Diabetes** (ICD-10) | 0 = no 1 = yes | **Inpatient Admissions + ED Presentations*** Denotes whether or not the patient had a primary or secondary diagnosis related to diabetes and were identified using ICD-10 codes. |
| **Neurological disease** (ICD-10) | 0 = no 1 = yes | **Inpatient Admissions + ED Presentations*** Denotes whether or not the patient had a primary or secondary diagnosis related to a neurological disease and were identified using ICD-10 codes. |
| **Number of screening blood tests** (out of 5) | Integer (possible values: 0,1,2,3,4,5) | **Inpatient Admissions + ED Presentations***  Total number of the following tests administered at hospital:   - Urea and Electrolytes (UEC) - Full Blood Examination (FBE) - Liver Function Tests (LFT) - Vitamin B12 blood test (B12) - Thyroid Stimulating Hormone (TSH) |
| **Psychosis** (ICD-10) | 0 = no 1 = yes | **Inpatient Admissions + ED Presentations*** Denotes whether or not the patient had a primary or secondary diagnosis related to a psychosis and were identified using ICD-10 codes. |
| **Allied health use** | 0 = no 1 = yes | **Inpatient Admissions + Community Health*** Denotes whether or not a patient used allied health services such as physical therapy, occupational therapy, speech pathology etc. |
| **Recorded interaction with residential aged care service** | 0 = no 1 = yes | **Inpatient Admissions + Community Health*** Denotes whether or not a patient used was either admitted from or discharged to locations/programs that would indicate a residential aged care facility (RACF) or participation in the Transition Care Program (TCP). |
| **Recorded use of APMH service** | 0 = no 1 = yes | **Inpatient Admissions + Outpatient*** Denotes whether or not the patient had a recorded history of receiving an Aged Persons Mental Health (APMH) service. |
| **Antidepressant medication use** | 0 = no 1 = yes | **Inpatient Admissions + ED Presentations + Outpatient*** Denotes whether or not the patient had a recorded history (including at discharge) of use of antidepressant medications (based on medication names listed in EHR). |
| **Cholinesterase inhibitor use** | 0 = no 1 = yes | **Inpatient Admissions + ED Presentations + Outpatient*** Denotes whether or not the patient had a recorded history (including at discharge) of use of cholinesterase inhibitors (based on medication names listed in EHR). |
| **CVD medication use** | 0 = no 1 = yes | **Inpatient Admissions + ED Presentations + Outpatient*** Denotes whether or not the patient had a recorded history (including at discharge) of use of cardiovascular disease-related medications (based on medication names listed in EHR). |
| **Depression medication use** | 0 = no 1 = yes | **Inpatient Admissions + ED Presentations + Outpatient*** Denotes whether or not the patient had a recorded history (including at discharge) of use of depression-related medications (based on medication names listed in EHR). |
| **Psychotropic medication use** | 0 = no 1 = yes | **Inpatient Admissions + ED Presentations + Outpatient*** Denotes whether or not the patient had a recorded history (including at discharge) of use of psychotropic medications (based on medication names listed in EHR). |
| **Recorded use of Imaging** (CT/MRI) | 0 = no 1 = yes | **Inpatient Admissions + ED Presentations + Outpatient*** Denotes whether or not the patient had a recorded history of receiving a Computed Tomography (CT) or Magnetic Resonance Imaging (MRI) scan. |
| **Neurologist Appointment Attendance** | 0 = no 1 = yes | **Inpatient Admissions + Community Health + Outpatient*** Denotes whether or not a patient was seen by a neurologist in any setting |

Abbreviations: APMH, Aged Persons Mental Health; CVD, cardiovascular disease; ED, emergency department; EHR, Electronic Health Record; RACF, residential aged care facility; TCP, Transition Care Program.

* Time-varying in data. Average date range for the dementia cohort was 22 May 2016 to 17th May 2021. The non-dementia cohort date range was Feb 6th 2016 to Aug 6th 2020 (inclusive).

## Supplementary Table 8: Predictors retained by LASSO

| **Predictor Retained by LASSO** | **Tier**(expert rating) | **Regularised Coefficient** (LASSO)^ |
| --- | --- | --- |
| Dementia ICD Code | 1 | 2.938 |
| Cholinesterase inhibitor use | 1 | 2.406 |
| Psychosis (ICD-10) | 2 | -0.84 |
| Behavioural Disturbance Codes (Code Grey) | 1 | 0.797 |
| Recorded interaction with residential aged care service | 1 | 0.683 |
| Neurological disease (ICD-10) | 2 | -0.609 |
| CVD medication use | 2 | -0.574 |
| Cardiovascular Disease (ICD-10) | 2 | -0.43 |
| Depression (ICD-10) | 2 | -0.318 |
| Neurology Outpatients Appointment | 1 | -0.313 |
| Cerebrovascular Disease (ICD-10) | 2 | 0.178 |
| Number of Emergency Department (ED) visits | 2 | -0.07 |
| Age | 1 | 0.063 |
| Female Sex | 2 | 0.0261 |
| Number of Outpatient visits | 2 | -0.02 |
| Index of Relative Socioeconomic Advantage and Disadvantage (IRSAD) | 2 | 0.001 |

## ^ LASSO = Least Absolute Shrinkage and Selection Operator. These coefficients should be interpreted with caution as they are biased due to regularization, and depend on units used. In general, however, they do provide insight into the relative magnitude and direction of each variable's effect on the outcome (in particular, note the substantial contribution of the Dementia ICD Code and Cholinesterase inhibitor use.)

## Supplementary Table 9: Sensitivity analysis excluding those scoring < average on TICS-M

|  | Models/Metrics | **AUC^** | **Classification**  **Threshold** | **% Correctly Classified (Accuracy)** | **Sensitivity (Recall)** | **Specificity** | **PPV  (Precision)** | **NPV** |  |
| --- | --- | --- | --- | --- | --- | --- | --- | --- | --- |
| Structured Data Modelling Stream  (Logistic Regression)   Development,  n= 989 | Age Only | 0.660 | 0.35 | 60.9% | 68.51 | 56.46 | 47.6 | 75.6 |  |
|  | Base Model | 0.799 | 0.25 | 69.4% | 76.80 | 65.07 | 55.9 | 82.9 |  |
|  | Full Model | 0.845 | 0.30 | 74.7% | 71.18 | 72.3 | 62.3 | 85.2 |  |
|  | LASSO-Penalised | 0.845 | 0.30 | 75.3% | 77.90 | 73.84 | 63.2 | 85.3 |  |
| NLP Modelling Stream    Development,  N= 773 | Keyword | 0.525 | - | 35.20% | 52.50 | 10.50 | 56.30 | 56.30 |  |
|  | Logistic Regression | 0.7962 | 0.4 | 82.94% | 79.62 | 80.93 | 89.18 | 80.93 |  |
|  | Naïve Bayes | 0.6660 | 0.5 | 69.28% | 66.60 | 65.71 | 74.07 | 65.71 |  |
|  | Support Vector Machine | 0.8015 | 0.4 | 83.92% | 80.15 | 82.49 | 91.03 | 82.49 |  |
|  | AdaBoost | 0.7866 | 0.5 | 82.62% | 78.66 | 80.59 | 89.86 | 80.59 |  |
|  | Random Forest | 0.8009 | 0.45 | 83.71% | 80.09 | 82.20 | 90.67 | 82.20 |  |

## Supplementary Table 10: ICD code sets

| **Condition** | **ICD diagnosis code substring (first 3 characters)** | **ICD diagnosis code substring (first 4 characters)** |
| --- | --- | --- |
| Neurological disease | B31, B32, B33, B34, G81, G82, G10, G11, G12, G13, G20, G21, G22, G32, G35, G36, G37, G40, G41, G43, R41, R56, U80 | G041, G114, G801, G802, G830, G831, G832, G833, G834, G839, G254, G255, G312, G318, G319, G931, G934, R470, |
| Depression | F32, F33, F34, F38, F39 | F063, F204, F313, F314, F315, F412, F432, U793, |
| Cardiovascular disease | First 2 characters ‘I8’  I47, I48, I49, I05, I06, I07, I08, I34, I35, I36, I37, I38, I39, I20, I21, I22, I23, I24, I25, I26, I27, I70, I71, I731, I738, I739, I771, I790, I792, K551, K558, K559, I10, I11, I12, I13, I15, I95, I97, I99 | I099, I130, I132, I420, I425, I426, I427, I428, I429, P290, I441, I442, I443, I456, I459, R000, R001, R008, T821, Z450, Z950, A520, I091, I098, Q230, Q231, Q232, Q233, Z952, Z953, Z954, I280, I288, I289, I988, Z958, Z959 |
| Diabetes | E10, E11, E13, E14 | E102, E103, E104, E105, E107, E112, E113, E114, E115, E117, E122, E123, E124, E125, E127 |
| Cerebrovascular disease (including traumatic brain injury) | G45, G46, I60, I61, I62, I63, I64, I65, I66, I67, I69, S04, S06 | I681, I682, I688, Z866, I673, S020, S021, S027, S028, S029, S071, S078, S079, S097, S099, T020, T040, T060 |
| Atrial fibrillation | I48 |  |
| Dementia | F00, F01, F02, F03, F04 | F000, F001, F002, F009, F010, F011, F012, F013, F018, F019, F020, F021, F021, F022, F023, F024, F028, F050, F051, F061, G300, G301, G308, G309, G311, R411, R412, R413, B63A, B63B, B63Z, U791  First 5 characters G3101, G3109, G3183, G3184 |

## Supplementary Table 11: Medication Lists

| **Category** | **Drug name** | **ATC codes** |
| --- | --- | --- |
| **Alzheimer's Disease-specific medications** | | |
|  | DONEPEZIL | N06DA02 |
|  | RIVASTIGMINE | N06DA03 |
|  | GALANTAMINE | N06DA04 |
|  | MEMANTINE | N06DX01 |
| **Antidepressants**   - **tricyclics** | | |
|  | DESIPRAMINE HYDROCHLORIDE | N06AA01 |
|  | IMIPRAMINE | N06AA02 |
|  | CLOMIPRAMINE | N06AA04 |
|  | TRIMIPRAMINE | N06AA06 |
|  | AMITRIPTYLINE | N06AA09 |
|  | DOXEPIN | N06AA12 |
|  | DOSULEPIN (DOTHIEPIN) | N06AA16 |
| **Antidepressants**   - others | | |
|  | FLUOXETINE | N06AB03 |
|  | CITALOPRAM | N06AB04 |
|  | PAROXETINE | N06AB05 |
|  | SERTRALINE | N06AB06 |
|  | FLUVOXAMINE | N06AB08 |
|  | ESCITALOPRAM | N06AB10 |
|  | PHENELZINE | N06AF03 |
|  | TRANYLCYPROMINE | N06AF04 |
|  | MOCLOBEMIDE | N06AG02 |
|  | MIANSERIN | N06AX03 |
|  | NEFAZODONE HYDROCHLORIDE | N06AX06 |
|  | MIRTAZAPINE | N06AX11 |
|  | VENLAFAXINE | N06AX16 |
|  | REBOXETINE | N06AX18 |
|  | DULOXETINE | N06AX21 |
|  | DESVENLAFAXINE | N06AX23 |
| **Benzodiazepines** (i.e. anxiety; does not include benzodiazepines used mainly as sedative) | | |
|  | DIAZEPAM | N05BA01 |
|  | CHLORDIAZEPOXIDE | N05BA02 |
|  | OXAZEPAM | N05BA04 |
|  | CLORAZEPATE DIPOTASSIUM | N05BA05 |
|  | LORAZEPAM | N05BA06 |
|  | BROMAZEPAM | N05BA08 |
|  | ALPRAZOLAM | N05BA12 |
| **Hypertension**   - **diuretics** | | |
|  | BENDROFLUAZIDE | C03AA01 |
|  | HYDROCHLOROTHIAZIDE | C03AA03 |
|  | CHLOROTHIAZIDE | C03AA04 |
|  | CYCLOPENTHIAZIDE | C03AA07 |
|  | METHYCLOTHIAZIDE | C03AA08 |
|  | QUINETHAZONE | C03BA02 |
|  | CHLORTALIDONE | C03BA04 |
|  | METOLAZONE | C03BA08 |
|  | INDAPAMIDE | C03BA11 |
|  | FUROSEMIDE (FRUSEMIDE) | C03CA01 |
|  | BUMETANIDE | C03CA02 |
|  | ETACRYNIC ACID | C03CC01 |
|  | AMILORIDE | C03DB01 |
|  | TRIAMTERENE | C03DB02 |
|  | DIURETIC COMBINATIONS | C03EA01 |
|  | TOLVAPTAN | C03XA01 |
| **Hypertension**  **- Beta blockers (usually not on their own for hypertension)** | | |
|  | ALPRENOLOL HYDROCHLORIDE | C07AA01 |
|  | OXPRENOLOL | C07AA02 |
|  | PINDOLOL | C07AA03 |
|  | PROPRANOLOL | C07AA05 |
|  | TIMOLOL | C07AA06 |
|  | METOPROLOL TARTRATE | C07AB02  (immediate release) |
|  | METOPROLOL SUCCINATE | C07AB02  (extended release) |
|  | ATENOLOL | C07AB03 |
|  | BISOPROLOL | C07AB07 |
|  | LABETALOL | C07AG01 |
|  | CARVEDILOL | C07AG02 |
| **Calcium channel blockers** (usually not on their own for hypertension) | | |
|  | AMLODIPINE | C08CA01 |
|  | FELODIPINE | C08CA02 |
|  | NIFEDIPINE | C08CA05 |
|  | LERCANIDIPINE | C08CA13 |
|  | MIBEFRADIL DIHYDROCHLORIDE | C08CX01 |
|  | VERAPAMIL | C08DA01 |
|  | DILTIAZEM | C08DB01 |
|  | PERHEXILINE | C08EX02 |
| **Ace-Inhibitors** | | |
|  | CAPTOPRIL | C09AA01 |
|  | ENALAPRIL | C09AA02 |
|  | LISINOPRIL | C09AA03 |
|  | PERINDOPRIL | C09AA04 |
|  | RAMIPRIL | C09AA05 |
|  | QUINAPRIL | C09AA06 |
|  | CILAZAPRIL MONOHYDRATE | C09AA08 |
|  | FOSINOPRIL | C09AA09 |
|  | TRANDOLAPRIL | C09AA10 |
| **Ace-Inhibitor combinations** | | |
|  | ENALAPRIL + HYDROCHLOROTHIAZIDE | C09BA02 |
|  | PERINDOPRIL + INDAPAMIDE | C09BA04 |
|  | QUINAPRIL + HYDROCHLOROTHIAZIDE | C09BA06 |
|  | FOSINOPRIL + HYDROCHLOROTHIAZIDE | C09BA09 |
|  | LERCANIDIPINE + ENALAPRIL | C09BB02 |
|  | PERINDOPRIL + AMLODIPINE | C09BB04 |
|  | RAMIPRIL + FELODIPINE | C09BB05 |
|  | TRANDOLAPRIL + VERAPAMIL | C09BB10 |
| **AT2 Inhibitors** | | |
|  | LOSARTAN | C09CA01 |
|  | EPROSARTAN | C09CA02 |
|  | VALSARTAN | C09CA03 |
|  | IRBESARTAN | C09CA04 |
|  | CANDESARTAN | C09CA06 |
|  | TELMISARTAN | C09CA07 |
|  | OLMESARTAN | C09CA08 |
| **AT2 Inhibitor combinations** | | |
|  | EPROSARTAN + HYDROCHLOROTHIAZIDE | C09DA02 |
|  | VALSARTAN + HYDROCHLOROTHIAZIDE | C09DA03 |
|  | IRBESARTAN + HYDROCHLOROTHIAZIDE | C09DA04 |
|  | CANDESARTAN + HYDROCHLOROTHIAZIDE | C09DA06 |
|  | TELMISARTAN + HYDROCHLOROTHIAZIDE | C09DA07 |
|  | OLMESARTAN + HYDROCHLOROTHIAZIDE | C09DA08 |
|  | AMLODIPINE + VALSARTAN | C09DB01 |
|  | OLMESARTAN + AMLODIPINE | C09DB02 |
|  | TELMISARTAN + AMLODIPINE | C09DB04 |
|  | AMLODIPINE + VALSARTAN + HYDROCHLOROTHIAZIDE | C09DX01 |
|  | OLMESARTAN + AMLODIPINE + HYDROCHLOROTHIAZIDE | C09DX03 |
|  | SACUBITRIL + VALSARTAN | C09DX04 |
| **Psychoactive drugs** (antipsychotics; does not include lithium) | | |
|  | CHLORPROMAZINE | N05AA01 |
|  | FLUPHENAZINE HYDROCHLORIDE | N05AB02 |
|  | TRIFLUOPERAZINE | N05AB06 |
|  | PERICIAZINE | N05AC01 |
|  | THIORIDAZINE | N05AC02 |
|  | HALOPERIDOL | N05AD01 |
|  | ZIPRASIDONE | N05AE04 |
|  | FLUPENTIXOL DECANOATE | N05AF01 |
|  | ZUCLOPENTHIXOL DECANOATE | N05AF05 |
|  | CLOZAPINE | N05AH02 |
|  | OLANZAPINE | N05AH03 |
|  | QUETIAPINE | N05AH04 |
|  | AMISULPRIDE | N05AL05 |
|  | RISPERIDONE | N05AX08 |
|  | ARIPIPRAZOLE | N05AX12 |
|  | PALIPERIDONE | N05AX13 |
|  | CARIPRAZINE | N05AX15 |
|  | BREXPIPRAZOLE | N05AX16 |
| **Sedative/hypnotic (i.e. sleep)** | | |
|  | FLURAZEPAM DIHYDROCHLORIDE | N05CD01 |
|  | NITRAZEPAM | N05CD02 |
|  | FLUNITRAZEPAM | N05CD03 |
|  | TEMAZEPAM | N05CD07 |
|  | MIDAZOLAM | N05CD08 |
|  | ZOPICLONE | N05CF01 |
| **Diabetes (insulin codes separate from others)** | | |
| **INSULINS** | INSULIN NEUTRAL HUMAN | A10AA01 |
|  | INSULIN ISOPHANE HUMAN | A10AA02 |
|  | INSULIN ISOPHANE HUMAN + INSULIN NEUTRAL HUMAN | A10AA03 |
|  | INSULIN PROTAMINE ZINC | A10AA04 |
|  | INSULIN NEUTRAL HUMAN | A10AB01 |
|  | INSULIN NEUTRAL BOVINE | A10AB02 |
|  | INSULIN LISPRO | A10AB04 |
|  | INSULIN ASPART | A10AB05 |
|  | INSULIN GLULISINE | A10AB06 |
|  | INSULIN ISOPHANE HUMAN | A10AC01 |
|  | INSULIN ZINC SUSPENSION (LENTE) | A10AC01 |
|  | INSULIN ISOPHANE HUMAN | A10AC01 |
|  | INSULIN ISOPHANE BOVINE | A10AC02 |
|  | INSULIN ISOPHANE HUMAN + INSULIN NEUTRAL HUMAN | A10AD01 |
|  | INSULIN LISPRO + INSULIN LISPRO PROTAMINE | A10AD04 |
|  | INSULIN ASPART + INSULIN ASPART PROTAMINE | A10AD05 |
|  | INSULIN DEGLUDEC + INSULIN ASPART | A10AD06 |
|  | INSULIN ZINC SUSPENSION (CRYSTALLINE) (ULTRALENTE) | A10AE01 |
|  | INSULIN ZINC SUSPENSION (CRYSTALLINE) (ULTRALENTE) | A10AE02 |
|  | INSULIN GLARGINE | A10AE04 |
|  | INSULIN DETEMIR | A10AE05 |
| **DIABETES - OTHERS** | **NON-INSULINS** |  |
|  | METFORMIN | A10BA02 |
|  | GLIBENCLAMIDE | A10BB01 |
|  | CHLORPROPAMIDE | A10BB02 |
|  | TOLBUTAMIDE | A10BB03 |
|  | TOLAZAMIDE | A10BB05 |
|  | GLIPIZIDE | A10BB07 |
|  | GLICLAZIDE | A10BB09 |
|  | GLIMEPIRIDE | A10BB12 |
|  | METFORMIN + GLIBENCLAMIDE | A10BD02 |
|  | ROSIGLITAZONE + METFORMIN | A10BD03 |
|  | SITAGLIPTIN + METFORMIN | A10BD07 |
|  | VILDAGLIPTIN + METFORMIN | A10BD08 |
|  | SAXAGLIPTIN + METFORMIN | A10BD10 |
|  | LINAGLIPTIN + METFORMIN | A10BD11 |
|  | ALOGLIPTIN + METFORMIN | A10BD13 |
|  | DAPAGLIFLOZIN + METFORMIN | A10BD15 |
|  | EMPAGLIFLOZIN + LINAGLIPTIN | A10BD19 |
|  | EMPAGLIFLOZIN + METFORMIN | A10BD20 |
|  | SAXAGLIPTIN + DAPAGLIFLOZIN | A10BD21 |
|  | ERTUGLIFLOZIN + METFORMIN | A10BD23 |
|  | ERTUGLIFLOZIN + SITAGLIPTIN | A10BD24 |
|  | ACARBOSE | A10BF01 |
|  | ROSIGLITAZONE | A10BG02 |
|  | PIOGLITAZONE | A10BG03 |
|  | SITAGLIPTIN | A10BH01 |
|  | VILDAGLIPTIN | A10BH02 |
|  | SAXAGLIPTIN | A10BH03 |
|  | ALOGLIPTIN | A10BH04 |
|  | LINAGLIPTIN | A10BH05 |
|  | SITAGLIPTIN + SIMVASTATIN | A10BH51 |
|  | EXENATIDE | A10BJ01 |
|  | DULAGLUTIDE | A10BJ05 |
|  | SEMAGLUTIDE | A10BJ06 |
|  | DAPAGLIFLOZIN | A10BK01 |
|  | EMPAGLIFLOZIN | A10BK03 |
|  | ERTUGLIFLOZIN | A10BK04 |
|  | CANAGLIFLOZIN | A10BX11 |

## Figure S1


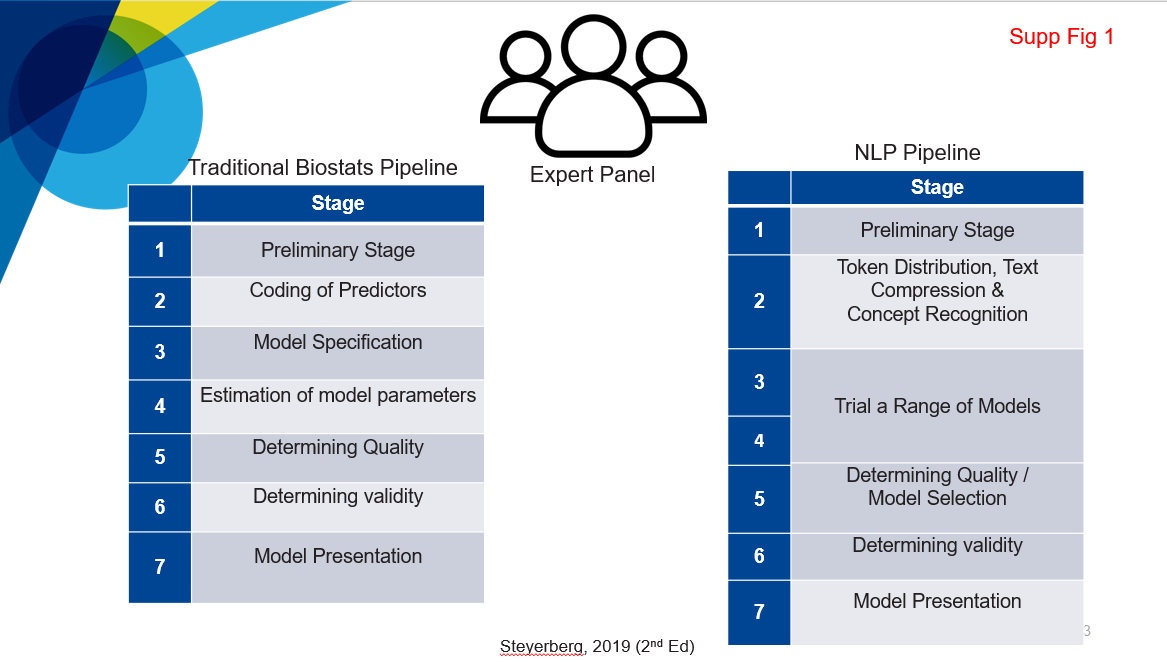

Supplement: Supplementary file 1 — Supporting Information [file ALZ-21-e70132-s003.docx]
